# Radiographic knee osteoarthritis severity has no impact on fall risk: the locomotive syndrome and health outcomes in the aizu cohort study (LOHAS): a cross-sectional study

**DOI:** 10.1186/s12891-024-07421-1

**Published:** 2024-04-16

**Authors:** Tatsuru Sonobe, Koji Otani, Miho Sekiguchi, Kenichi Otoshi, Takuya Nikaido, Mari Sato, Shinichi Konno, Yoshihiro Matsumoto

**Affiliations:** 1 Department of Orthopaedic Surgery, Fukushima Medical University School of Medicine, 1 Hikarigaoka, Fukushima-Shi, Fukushima, 960-1295 Japan; 2 Department of Sports Medicine, Fukushima Medical University School of Medicine, Fukushima, 960-1295 Japan; 3 Department of Rehabilitation Medicine, Fukushima Medical University School of Medicine, Fukushima, 960-1295 Japan

**Keywords:** Fall, Radiographic knee osteoarthritis, Knee pain, Psychological factor, Mobility, Timed up and go test

## Abstract

**Background:**

To investigate factors that have an impact on the risk of falls and determine whether radiographic knee osteoarthritis (KOA) is a factor involved in falls independent of knee pain, psychological factors, and physical function.

**Methods:**

A cross-sectional analysis was conducted on 1083 subjects for the 2009 Locomotive Syndrome and Health Outcomes in the Aizu Cohort Study (LOHAS). A logistic regression analysis was performed to examine the relationship between radiographic KOA and fall history.

**Results:**

Fall history was significantly associated with the severity of knee pain. Compared to subjects with no knee pain, the odds ratio (OR) was 1.53 times higher in the subjects with mild knee pain (95% confidence interval [CI]: 1.04–2.25), 1.69 times higher in those with moderate knee pain (95%CI: 1.03–2.79), and 2.98 times higher in those with severe knee pain (95%CI: 1.67–5.30). In subjects with depression, the OR was 1.91 (95%CI: 1.25–2.92), and in those with decreased mobility, the OR was 1.70 (95%CI: 1.08–2.69). Age, gender, knee crepitus, BMI, OLST, and sleeping pill use were not significantly associated with fall risk. In a multivariate analysis, radiographic KOA severity was not significantly associated with fall risk (OR 0.81, 95%CI 0.44–1.50 in mild OA; OR 1.10, 95%CI 0.57–2.14 in severe OA).

**Conclusion:**

Knee pain, decreased mobility, and depression, but not the radiographic KOA severity, were significantly associated with a fall risk. Regardless of the individual's radiographic KOA severity, the risk of falls may be reduced by treating his/her knee pain, mobility problems, and/or psychological factors.

## Background

Falls are a leading cause of injury-related morbidity and mortality among older adults, with more than one in four older adults falling each year [1]. Costs of care after falls in the elderly are burdensome, reaching over $31 billion dollars in the United States in 2015 [2]. Falls may also lead to a fear of falling, decreased physical function, limited physical activity, loss of independence, and a higher mortality risk [3, 4].

Knee osteoarthritis (KOA) is a major public health problem associated with aging in musculoskeletal disorders [5]. The prevalence of radiographic KOA in adults aged ≥ 60 years in the U.S. was estimated to be 37% [6]. Common symptoms of KOA include knee pain, stiffness, swelling, decreased mobility, muscle weakness, and difficulty walking and climbing stairs [7]. Pain, dysfunction, and disability are caused by progressive loss of articular cartilage [8] and synovitis [9] of the knee joint, and the progression of KOA tends to lead to increased physical limitations, pain, and functionality restrictions [10].

An association between KOA and the risk of falls has been reported in cross-sectional studies [11–14], prospective cohorts [15–18], and longitudinal studies [19–23] (Table 1), but the results are inconsistent and controversial [17]. Although the diagnosis of KOA is often based on the combination of plain radiographs and subjective symptoms, several studies have described the involvement of psychological factors such as depression in the severity of symptoms and physical function in patients with KOA [24]. The need to consider psychological factors in assessing the association between KOA and falls has been addressed [13], but we found no published investigation of the impact of radiographic KOA severity on fall risk that also takes into account psychological factors. Even with the same radiographic KOA severity, pain and physical function vary from case to case. Prior studies have shown that the radiographic KOA severity itself is associated with falls [14, 15, 20]. By considering a variety of confounding factors, including psychological factors that have not been examined previously, this would be a new finding to determine whether the radiographic KOA severity itself is a risk for falls. Furthermore, if the radiographic KOA severity itself is not a risk for falls, this finding sends an important clinical message that falls can be prevented by improving factors other than the radiographic KOA severity, which are difficult to improve with current medicine. We conducted the present study to (*i*) investigate factors that have an impact on the risk of falls, and (*ii*) clarify the impact of the radiographic KOA severity on the risk of falls.

**Table 1:** Summary of reviewed studies

First author, yr [ref.]	Participants	Study design	Define of KOA	Radiographic KOA	Correlation or risk	Main findings
Foley 2006 [11]	Total n = 850 Mean age 62.5 Age range ± 7.4	A	OARSI atlas	No significant association	Symptom, pain	Alleviation of musculoskeletal symptoms may lessen the risk of falls in older people
Muraki 2011 [12]	Total *n* = 1,675 Mean age 65.3 Age range ± 12.0	A	KL grade ≥ 3	No significant association	Women with pain	Knee pain was significantly associated with multiple falls in women
Mat 2011 [13]	Total *n* = 850 Mean age 62.5 Age range ± 7.10	A	Self-report, clinician diagnosis, KL grade ≥ 2	No significant association	Symptoms	Radiological OA with mild overall symptoms measured with the WOMAC score may be predictive of falls
Khalaj 2014 [14]	Total *n* = 60 Age range 50–70	A	Bilateral KL grade 2 or 3	Significant association	KL grade ≥ 2, dynamic and static balance	Bilateral knee osteoarthritis impaired balance and increased the risk of fall, particularly in people with moderate knee osteoarthritis
Harris 2023 [15]	Total *n* = 3972 Mean age 62.5 Age range ± 7.12	B	KL grade 0–4	Significant association	KL grade ≥ 1 (Age ≥ 65), KL grade ≥ 2 (Age < 65)	Older adults with radiographic evidence of KOA have an increased likelihood of experiencing recurrent falls in comparison to those without KOA independent of established risk factors
Doré 2015 [16]	Total *n* = 1,619 Mean age 62.0 Age range 45–89	B	KL grade ≥ 2	No significant association	Symptoms	The risk for falls increases with additional symptomatic OA lower limb joints; symptomatic hip and knee OA are important risk factors for falls
Cai 2022 [17]	Total *n* = 4,465 Mean age 61.2 Age range ± 9.2	B	KL grade ≥ 2	No significant association	Symptoms	Knee symptoms but not radiographic KOA increased the risk of falls, recurrent falls, and fractures
Scott 2012 [18]	Total *n* = 709 Mean age 62.0 Age range ± 7.0	B	OARSI atlas	No significant association	Women with pain, stiffness, dysfunction	Knee pain may directly contribute to the progression of sarcopenia and increased fall risk in older women
Barbour 2019 [19]	Total *n* = 734 Mean age 74.7 Age range ± 2.9	C	KL grade ≥ 2	No significant association	Men with symptom	Knee symptomatic radiographic OA was independently associated with a 2.6-fold increased risk of incident injurious falls in men only
Tsonga 2011 [20]	Total *n* = 68 Mean age 73.0 Age range ± 5.3	C	KL grade ≥ 3	Significant association with severe KOA	Pain, stiffness, limited physical ability, reduced muscle strength	Patients with severe knee OA were at greater risk of falling, as compared to healthy older adults
Arden 2006 [21]	Total *n* = 6641 Age ≥ 75 Age ≥ 75	C	Self-report	N/A	Pain	Knee pain and OA should be regarded as independent risk factors for fracture
van Schoor 2020 [22]	Total *n* = 2,535 Age range 65–85	C	ACR clinical classification criteria	No significant association	Pain, stiffness, crepitus, tenderness	Individuals with clinical knee OA were at increased risk for recurrent falls
Prieto-Alhambra 2012 [23]	Total *n* = 51,386 Age ≥ 55 Only women	C	Self-report	N/A	Self-report KOA	Interventions to reduce falls might be useful in preventing fractures in patients with osteoarthritis

*A* Cross-sectional study, *B* Prospective cohort, *C* Observational longitudinal study, *ACR* American College of Rheumatology, *KL* Kellgren-Lawrence, *KOA* Knee osteoarthritis, N/A Not available, *OA* Osteoarthritis, *OARSI* Osteoarthritis Research Society International, *WOMAC* Western Ontario and McMaster Universities Osteoarthritis Index

## Methods

### Participants

**Fig. 1:**
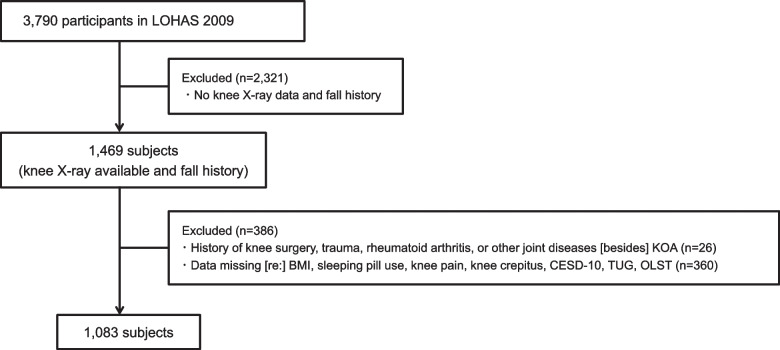
Flowchart of the subjects' enrollment. A total of 1,083 subjects were included in the analysis. LOHAS, the Locomotive Syndrome and Health Outcome in Aizu Cohort Study, BMI Body mass index, CESD-10 Center of Epidemiologic Studies Depression Scale-10, TUG Timed Up and Go Test, OLST One-Leg Standing test

The Aizu Cohort Study on Locomotive Syndrome and Health Outcomes (LOHAS) was designed in 2008 to investigate the quality of life (QOL), motor function, medical costs, mortality, and cardiovascular disease attributable to motor dysfunction. Residents of the Japanese towns of Minamiaizu and Tadami in Fukushima Prefecture who participated in annual health checkups conducted by their local governments were enrolled in the LOHAS. All of the participants were enrolled in the National Health Insurance System of Japan. The detailed design of LOHAS has been described [25]. The present study was a cross-sectional analysis using data from the 2009 LOHAS baseline survey, which included 3,790 participants, among whom all 1,469 subjects who requested the examination underwent plane radiography of both knee joints while in a standing position and responded to fall history. The 26 subjects with a history of knee surgery, trauma, rheumatoid arthritis, or joint diseases other than KOA were excluded from the present study, as were the 386 subjects with missing data regarding BMI, sleeping pill use, knee pain, knee crepitus, Center of Epidemiologic Studies Depression Scale-10 (CESD-10) score, Timed Up and Go test (TUG), One-leg Standing Test (OLST), or fall history. A final total of 1,083 subjects were included in the present analyses (Fig. 1).

Written informed consent for the use of data collected in LOHAS was obtained from all participants upon enrollment. The present study complied with the Declaration of Helsinki and was approved by the Research Ethics Committee of Fukushima Medical University (No. 295, 673).

The questionnaire used in LOHAS recorded the participant's occupation, employment status, surgical history, sleeping pill use, CESD-10 score, and fall history. Sleeping pill use is one of the most common causes of falls [26]. In this study, a history of sleeping pill use was defined as using sleeping pills at least once a week. The Center of Epidemiologic Studies Depression Scale (CES-D) is a self-reported psychometric scale intended to identify the frequency and severity of depressive symptoms [27]. The CESD-10 is a short form (10 items) of the CES-D that provides a simple assessment of depression; scores of ≥ 10 points indicate the presence of clinical depression [28]. We defined a fall as 'an event in which an individual unexpectedly comes to rest on the ground, floor, or lower level by tripping or stepping off.' The participants were asked 'Have you had any falls in the past 12 months?'. If the participant had a fall history, we defined him or her as “positive” for fall experiences.

### Knee symptoms

During the annual physical checkup, the degree of knee pain in the last month was recorded using a six-point scale (0 points, a complete absence of pain, 1 point; very slight pain, 2 points; slight pain; 3 points, moderate pain; 4 points, difficult pain; 5 points, severe pain) based on the participant's questionnaire response. Subjects who had any fall in the past month recorded regular knee pain after falls. If there was a fall history, the degree of knee pain prior to the fall was recorded. In our present analyses, knee pain was classified into four categories; 0 points, no pain; 1 or 2 points, mild pain; 3 points, moderate pain; and 4 or 5 points, severe pain.

The presence or absence of crepitus was also recorded. Crepitus is one of the physical symptoms of KOA. If crepitus was present in either knee as a physical symptom by self-report, the subjects were defined to have crepitus. Individuals with KOA and knee crepitus have been reportedly associated with falls [22]; therefore, knee crepitus was included in this analysis.

### Radiological assessment

The subjects' radiographic KOA severity was graded based on the Kellgren-Lawrence classification (KL grade) [29]. Two well-trained knee surgeons assessed the anterior–posterior view of both knee plane radiographs of subjects in the standing position. The intra-observer reliability was 0.653, and the inter-observer reliability was 0.652. Since the reported intra- and inter-observer reliability values of the KL-grade classification are 0.56 and 0.61, respectively, our grading accuracy equals or surpasses that of an earlier study [30]. When the knee surgeons' assessment of the KL grade for a subject did not match, consensus was reached via discussion. In the present study, we thus used the KL grade on the subject's more severe side and categorized the KL grades 0 as no OA, grades 1 and 2 as mild OA, and grades 3 and 4 as severe OA.

### Physical function

In the present study, we evaluated the subjects' physical function based on their TUG and OLST results. TUG evaluates mobility capability, and OLST assesses static balance. The TUG score is the time taken to get up from a chair without armrests, walk 3 m, return to the chair and sit down. Several studies have considered a TUG time ≥ 10 s to be the cutoff value for fall risk [31, 32]. In the present study we likewise defined a TUG time < 10 s as normal mobility and ≥ 10 s as decreased mobility. The OLST score is the length of time that a subject is able to stand on one leg (either side) with open eyes. An OLST time < 10 s is considered a useful predictor of fall risk in the elderly [33, 34]. We used the OLST score measured on the subject's side with the more severe KL grade in the present analyses, and we thus defined OLST times ≥ 10 s as normal balance and < 10 s as balance disability.

### Statistics analysis

Only the LOHAS subjects with complete data were enrolled in the primary analysis. Descriptive statistics were calculated for the subjects' baseline characteristics. Continuous data are summarized as means and standard deviations, and dichotomous or categorical data are presented as proportions. Means of continuous variables for the two groups were compared by t-test. To observe correlations between variables, Pearson's correlation coefficients were obtained. We performed univariate and multivariate logistic analyses to investigate the association between fall history and several factors, and we used a categorical variable, i.e., fall history, as the dependent variable. The categorical variables age, gender, BMI, sleeping pill use, knee pain, knee crepitus, CESD-10, TUG, OLST, and radiographic KOA severity were used as independent variables. In addition, we also performed a multivariate analysis excluding subjects with no OA, to consider for depression and decreased mobility not associated with KOA. Variance inflation factor (VIF) is a measure of multicollinearity in multivariate analysis. A high VIF indicates that the associated independent variable is highly collinear with other variables in the model. The odds ratios (OR) and 95% confidence intervals (CI) were calculated. All tests of statistical significance were two-tailed. Probability (p)-values < 0.05 were considered significant. All analyses were conducted using JMP PRO 16 (SAS, Cary, NC, USA).

## Results

### The subjects' characteristics

The characteristics of the 1,083 subjects are summarized in Table 2. Compared to the excluded subjects, the enrolled subjects tended to be older and more likely to use sleeping pills, have severe knee pain and crepitus, have decreased mobility, and be a positive for falls.

**Table 2:** Subject characteristics

	**All subjects** *n* **= 3,790**	**Excluded subjects n = 2,707**	**Analyzed subjects** ***n* = 1,083**	**Probability (*p*)-value**
Age, yrs: mean (95%CI)	67.5 (58.4–76.5)	66.9 (57.5–76.3)	68.7 (60.9–76.5)	** < 0.0001**
Age, yrs:				** < 0.0001**
< 65	1200 (31.6)	938 (34.7)	261 (24.1)	
65–74	1640 (43.3)	1085 (40.1)	555 (51.2)	
≥ 75	950 (25.1)	683 (25.2)	267 (24.7)	
Gender:				0.8707
Male	1560 (41.2)	1112 (41.1)	448 (41.4)	
Female	2230 (58.8)	1595 (58.9)	635 (58.6)	
BMI:				0.2109
< 18.5	98 (3.1)	70 (3.4)	28 (2.6)	
18.5–24.9	1981 (63.6)	1307 (64.3)	674 (62.2)	
25.0–29.9	921 (29.6)	579 (28.5)	342 (31.6)	
≥ 30	116 (3.7)	77 (3.8)	39 (3.6)	
Sleeping pill use:				**0.0326**
Absence	3231 (86.3)	2317 (87.1)	914 (84.4)	
Presence	513 (13.7)	344 (12.9)	169 (15.6)	
Knee pain:				** < 0.0001**
No pain	1155 (30.5)	1378 (50.9)	419 (38.7)	
Mild pain	425 (11.2)	906 (33.5)	419 (38.7)	
Moderate pain	196 (5.2)	268 (9.9)	157 (14.5)	
Severe pain	47 (1.2)	155 (5.7)	88 (8.1)	
Knee crepitus				**0.0048**
Absence	3139 (87.3)	1776 (73.4)	745 (68.8)	
Presence	455 (12.7)	642 (26.6)	338 (31.2)	
CESD-10:				0.1318
Not depressive	3139 (87.3)	2207 (87.9)	932 (86.1)	
Depressive	455 (12.7)	304 (12.1)	151 (13.9)	
TUG:				** < 0.0001**
Normal mobility	2978 (84.4)	2025 (82.9)	953 (88.0)	
Decreased mobility	549 (15.6)	419 (17.1)	130 (12.0)	
OLST:				0.9602
Normal balance	2923 (83.6)	2017 (83.6)	906 (83.7)	
Balance disability	573 (16.4)	396 (16.4)	177 (16.3)	
Fall history:				**0.0027**
No fall	3127 (83.4)	2255 (84.6)	872 (80.5)	
Positive fall experience	622 (16.6)	411 (15.4)	211 (19.5)	

The data are n (%). *BMI* Body mass index, *CESD-10* Center of Epidemiologic Studies Depression Scale-10, *TUG* Timed Up and Go Test, *OLST* One-Leg Standing Test

### The prevalence and grade classification of knee pain

The degree and classification of knee pain in the 1,083 subjects were as follows: no pain in 419 subjects (38.7%), mild pain (1 or 2 points) in 419 subjects (38.7%), moderate pain in 157 subjects (14.5%), and severe pain (4 or 5 points) in 88 subjects (8.1%).

### The prevalence of KL-grade more severe side and radiographic KOA severity

The prevalence of the KL-grade severe side (i.e., whether the side with the more severe KL grade was the left or right side) and the radiographic KOA severity are summarized in Table 3.

**Table 3:** The prevalence of the KL-grade more severe side and the radiographic KOA severity

KL grade		Radiographic	
		**KOA severity**	
0	83 (7.7)	No OA	83 (7.7)
1	322 (29.7)	Mild OA	619 (57.2)
2	297 (27.4)		
3	293 (27.1)	Severe OA	381 (35.1)
4	88 (8.1)		

The data are n (%). *KL* Kellgren-Lawrence, *KOA* Knee osteoarthritis, *OA* Osteoarthritis

### Association between fall history and continuous variables in T-test

In the T-test, CESD-10 and TUG were significantly higher in the positive fall experience group. Conversely, OLST were significantly lower in the positive fall experience group (Table 4).

**Table 4:** T-test for falls

Fall history	No fall *n* = 879	Positive fall experience *n* = 213	Probability (*p*)-value
CESD-10: mean ± SD	4.9 ± 4.0	6.3 ± 4.4	** < 0.0001**
TUG: mean ± SD	7.7 ± 2.0	8.4 ± 3.1	** < 0.0001**
OLST: mean ± SD	23.5 ± 9.0	19.7 ± 10.4	** < 0.0001**

### Factors associated with fall history in univariate analysis

In univariate analyses, significant associations were found in age ≥ 75 years (OR 1.66, 95%CI: 1.08–2.56 compared to the subjects aged < 65 years), knee pain (OR 1.63, 95%CI 1.12–2.35 in mild knee pain, OR 2.00, 95%CI 1.26–3.18 in moderate knee pain, OR 4.08, 95%CI 2.44–6.82 in severe knee pain, compared to the subjects with no knee pain), depression (OR 2.20, 95%CI 1.50–3.22), decreased mobility (OR 2.51, 95%CI 1.69–3.74), and balance disability (OR 2.02, 95%CI 1.41–2.91) (Table 5).

**Table 5:** Univariate analysis for falls

Fall history	Crude analysis	
	**OR (95%CI)**	**Probability (*p*)-value**
Age, yrs:		
< 65 years	ref	ー
65–74 years	1.14 (0.77–1.69)	0.5073
≥ 75 years	**1.66** (1.08–2.56)	**0.0200**
Gender:		
Male	ref	–
Female	1.29 (0.94–1.76)	0.1097
BMI:		
< 18.5	1.52 (0.63–3.66)	0.3472
18.5–24.9	ref	–
25.0–29.9	1.35 (0.98–1.86)	0.0660
≥ 30	0.67 (0.26–1.75)	0.4168
Sleep pill use:		
Absence	ref	–
Presence	1.19 (0.80–1.78)	0.3895
Knee pain:		
No	ref	–
Mild	**1.63** (1.12–2.35)	**0.0099**
Moderate	**2.00** (1.26–3.18)	**0.0034**
Severe	**4.08** (2.44–6.82)	** < 0.0001**
Knee crepitus:		
Absence	ref	–
Presence	1.24 (0.91–1.71)	0.1778
CESD–10:		
Not depressive	ref	–
Depressive	**2.20** (1.50–3.22)	** < 0.0001**
TUG:		
Normal mobility	ref	–
Decreased mobility	**2.51** (1.69–3.74)	** < 0.0001**
OLST:		
Normal balance	ref	–
Balance disability	**2.02** (1.41–2.91)	**0.0002**
Radiographic KOA severity:		
No OA	ref	–
Mild OA	0.86 (0.47–1.57)	0.6299
Severe OA	1.55 (0.85–2.83)	0.1565

In the univariate analysis, a logistic regression analysis was performed with age, gender, BMI, sleeping pill use, knee pain, knee crepitus, CESD-10, TUG, OLST, and radiographic KOA severity, respectively

*BMI* Body mass index, *CESD–10* Center of Epidemiologic Studies Depression Scale–10, *TUG* Timed Up and Go Test, *OLST* One-Leg Standing Test, *KOA* Knee osteoarthritis, *OA* Osteoarthritis

### Factors associated with fall history in multivariate analysis about KOA

The relationship between confounders (age, gender, BMI, sleeping pill use, knee pain, knee crepitus, CESD-10, TUG, OLST, and radiographic KOA severity [excluding no OA: excluding no OA pattern]) and fall history is shown in Table 6. Patterns excluding groups included in the no OA category in the radiographic KOA severity were defined as excluding no OA pattern. In the multivariate analysis, fall history was significantly associated with knee pain. Compared to subjects with no pain, the OR was 1.53 times higher in subjects with mild pain (95%CI: 1.04–2.25), 1.69 times higher in those with moderate pain (95%CI: 1.03–2.79), and 2.98 times higher in those with severe pain (95%CI: 1.67–5.30); thus, the OR increased as the pain worsened.

**Table 6:** Multivariate analysis for falls about KOA

Fall history	Multivariate analysis			Multivariate analysis: excluding no OA pattern		
	**OR (95%CI)**	**Probability (*p*)-value**	**VIF**	**OR (95%CI)**	**Probability (*p*)-value**	**VIF**
Age, yrs:						
< 65 years	ref	ー	1.26	ref	ー	1.26
65–74 years	0.97 (0.64–1.47)	0.8759		0.98 (0.63–1.54)	0.9399	
≥ 75 years	1.14 (0.69–1.89)	0.6131		1.17 (0.69–2.00)	0.5574	
Gender:						
Male	ref	–	1.11	ref	–	1.12
Female	1.06 (0.76–1.50)	0.7197		1.04 (0.72–1.48)	0.8466	
BMI:						
< 18.5	1.48 (0.57–3.79)	0.4184	1.07	1.35 (0.49–3.73)	0.5673	1.07
18.5–24.9	ref	–		ref	–	
25.0–29.9	1.16 (0.83–1.62)	0.404		1.26 (0.89–1.78)	0.1999	
≥ 30	0.51 (0.19–1.39)	0.1896		0.52 (0.19–1.41)	0.1963	
Sleeping pill use:						
Absence	ref	–	1.14	ref	–	1.15
Presence	0.71 (0.45–1.12)	0.1412		0.63 (0.39–1.27)	0.0555	
Knee pain:						
No	ref	–	1.23	ref	–	1.23
Mild	**1.53 (1.04–2.25)**	**0.0310**		**1.58** (1.05–2.37)	**0.0310**	
Moderate	**1.69 (1.03–2.79)**	**0.0389**		**1.78** (1.03–3.07)	**0.0381**	
Severe	**2.98 (1.67–5.30)**	**0.0002**		**2.81** (1.55–5.30)	**0.0007**	
Knee crepitus:						
Absence	ref	–	1.18	ref	–	1.19
Presence	0.84 (0.59–1.22)	0.3611		0.88 (0.60–1.27)	0.4912	
CESD–10:						
Not depressive	ref	–	1.13	ref	–	1.13
Depressive	**1.91 (1.25–2.92)**	**0.003**		**2.11** (1.36–3.29)	**0.0009**	
TUG:						
Normal mobility	ref	–	1.17	ref	–	1.18
Decreased mobility	**1.70 (1.08–2.69)**	**0.0224**		**1.64** (1.03–2.61)	**0.0385**	
OLST:						
Normal balance	ref	–	1.18	ref	–	1.18
Balance disability	1.35 (0.89–2.06)	0.161		1.49 (0.97–2.29)	0.0708	
Radiographic KOA severity:						
No OA	ref	–	1.29	–	–	1.31
Mild OA	0.81 (0.44–1.50)	0.5076		ref	–	
Severe OA	1.10 (0.57–2.14)	0.77		1.33 (0.92–1.94)	0.1305	

In the multivariate analysis, a logistic regression analysis was performed with age, gender, BMI, sleeping pill use, knee pain, knee crepitus, CESD–10, TUG, OLST, and radiographic KOA severity (excluding no OA: excluding no OA pattern) as covariates

*KOA* Knee osteoarthritis, *BMI* Body mass index, *CESD–10* Center of Epidemiologic Studies Depression Scale–10, *TUG* Timed Up and Go Test, *OLST* One-Leg Standing Test, *OA* Osteoarthritis

In subjects with depression, the OR was 1.91 (95%CI: 1.25–2.92). Depression was also significantly associated with fall history. Moreover, in subjects with decreased mobility, the OR was 1.70 (95%CI: 1.08–2.69), and in subjects with balance disability, the OR was 1.35 (95%CI: 0.89–2.06). Decreased mobility evaluated by TUG was thus significantly associated with fall history, while balance disability evaluated by OLST was not. Age, gender, BMI, sleeping pill use, and knee crepitus were not significantly associated with decreased mobility in multivariate analysis. The results of this study demonstrated that the radiographic KOA severity was not significantly associated with subjects’ fall history (OR: 0.81, 95%CI: 0.44–1.50 in mild OA, OR: 1.10, 95%CI: 0.57–2.14 in severe OA). As the VIF of each covariate was quite low in this analysis, there was no multicollinearity between the covariates.

In the excluding no OA pattern, significant associations were found in knee pain (OR 1.58, 95%CI 1.05–2.37 in mild knee pain, OR 1.78, 95%CI 1.03–3.07 in moderate knee pain, OR 2.81, 95%CI 1.55–5.30 in severe knee pain, compared to the subjects with no knee pain), depression (OR 2.11, 95%CI 1.36–2.61), decreased mobility (OR 1.64, 95%CI 1.03–2.61). As the VIF of each covariate was quite low in this analysis, there was no multicollinearity between the covariates.

### Factors associated with fall history in multivariate analysis about physical function

The relationship between confounders (age, gender, BMI, sleeping pill use, knee pain, knee crepitus, CESD-10, [TUG: excluding OLST pattern], [OLST: excluding TUG pattern], and radiographic KOA severity) and fall history is shown in Table 7. In excluding OLST pattern, significant associations were found in knee pain (OR 1.51, 95%CI 1.03–2.22 in mild knee pain, OR 1.70, 95%CI 1.03–2.80 in moderate knee pain, OR 3.11, 95%CI 1.76–5.52 in severe knee pain, compared to the subjects with no knee pain), depression (OR 1.95, 95%CI 1.27–2.97), decreased mobility (OR 1.79, 95%CI 1.14–2.80). In excluding TUG pattern, significant associations were found in knee pain (OR 1.53, 95%CI 1.04–2.25 in mild knee pain, OR 1.76, 95%CI 1.07–2.88 in moderate knee pain, OR 3.11, 95%CI 1.75–5.51 in severe knee pain, compared to the subjects with no knee pain), depression (OR 2.01, 95%CI 1.32–3.05).

**Table 7:** Multivariate analysis for fall about physical function

Fall history	Multivariate analysis: excluding OLST pattern			Multivariate analysis: excluding TUG pattern		
	**OR (95%CI)**	**Probability (*p*)-value**	**VIF**	**OR (95%CI)**	**Probability (*p*)-value**	**VIF**
Age, yrs:						
< 65 years	ref	ー	1.21	ref	ー	1.21
65–74 years	0.98 (0.65–1.49)	0.9370		0.99 (0.65–1.50)	0.9521	
≥ 75 years	1.22 (0.74–2.00)	0.4286		1.29 (0.79–2.11)	0.3114	
Gender:						
Male	ref	–	1.11	ref	–	1.11
Female	1.08 (0.77–1.52)	0.6426		1.07 (0.76–1.51)	0.6923	
BMI:						
< 18.5	1.53 (0.60–3.91)	0.3737	1.06	1.51 (0.59–3.83)	0.3876	1.07
18.5–24.9	ref	–		ref	–	
25.0–29.9	1.18 (0.84–1.65)	0.3392		1.16 (0.83–1.62)	0.3967	
≥ 30	0.54 (0.20–1.45)	0.2209		0.52 (0.19–1.41)	0.1976	
Sleeping pill use:						
Absence	ref	–	1.15	ref	–	1.15
Presence	0.72 (0.45–1.13)	0.1552		0.72 (0.46–1.14)	0.1642	
Knee pain:						
No	ref	–	1.22	ref	–	1.21
Mild	**1.51** (1.03–2.22)	**0.0345**		**1.53** (1.04–2.25)	**0.0294**	
Moderate	**1.70** (1.03–2.80)	**0.0373**		**1.76** (1.07–2.88)	**0.0266**	
Severe	**3.11** (1.76–5.52)	**0.0001**		**3.11** (1.75–5.51)	**0.0001**	
Knee crepitus:						
Absence	ref	–	1.18	ref	–	1.18
Presence	0.86 (0.60–1.24)	0.4152		0.86 (0.60–1.24)	0.4176	
CESD–10:						
Not depressive	ref	–	1.13	ref	–	1.12
Depressive	**1.95** (1.27–2.97)	**0.0021**		**2.01** (1.32–3.05)	**0.0012**	
TUG:						
Normal mobility	ref	–	1.15	–	–	–
Decreased mobility	**1.79** (1.14–2.80)	**0.0114**		–	–	
OLST:						
Normal balance	–	–	–	ref	–	1.14
Balance disability	–	–		1.45 (0.96–2.20)	0.0767	
Radiographic KOA severity:						
No OA	ref	–	1.29	ref	–	1.29
Mild OA	0.81 (0.44–1.49)	0.4936		0.83 (0.45–1.53)	0.5540	
Severe OA	1.10 (0.57–2.14)	0.7677		1.15 (0.59–2.22)	0.6874	

In the multivariate analysis without no OA, a logistic regression analysis was performed with age, gender, BMI, sleeping pill use, knee pain, knee crepitus, CESD–10, (TUG: excluding OLST pattern), (OLST: excluding TUG pattern), and radiographic KOA severity as covariates

*BMI* Body mass index, *CESD–10* Center of Epidemiologic Studies Depression Scale–10, *TUG* Timed Up and Go Test, *OLST* One-Leg Standing Test, *KOA* Knee osteoarthritis, *OA* Osteoarthritis

### Correlation between knee pain and depression

The correlation coefficient between knee pain and depression was 0.2, with a probability (*p*)-value of < 0.0001.

## Discussion

Our findings revealed that knee pain, decreased mobility, and depressive mood were significantly associated with a fall history. On the other hand, the radiographic KOA severity was not associated with a fall history.

Osteoarthritis has been considered an established risk factor for falls [35]. Regarding KOA, several studies [11–23] have obtained inconsistent results, based on different diagnoses and classifications of KOA. We analyzed the influence of radiographic KOA itself on the risk of falls, including as covariates factors that have been reported as a correlation or risk factor for falls (i.e., gender, pain, limited physical ability, and imbalance)^11–23^ and a psychological factor (depression) [13]. Falls exacerbate knee pain [20]. In this study, to minimize the impact of falls on regular knee pain, we assessed knee pain after the falls. Our present investigation is the first to analyze the impact of radiographic KOA on falls, taking into account not only the symptoms of KOA but also psychological factors and physical function. Our results corroborate and extend the prior studies' findings.

According to the present results, having a fall history was significantly associated with knee pain. Knee pain and other symptoms have been reported to be risk factors for falls among individuals with KOA [11, 12, 18, 20–22], and knee pain was significantly associated with falls in the present study as well. Pain related to OA leads to decreased use of the affected limbs and joints, muscle weakness, and poor functional performance, resulting in an increased risk of falls [16]. However, in KOA, the degree of knee pain is not necessarily related to radiographic severity [36]. In addition, we detected no significant association between radiographic KOA severity and fall history in a multivariate logistic analysis. In clinical practice, the radiographic severity tends to be more focused on the diagnosis of KOA, but we must not forget to properly evaluate knee pain when assessing its relationship to falls. Regardless of the severity of the imaging findings, the treatment of knee pain may improve an individual's risk of falling.

Our analyses revealed that decreased mobility was significantly associated with fall history. Decreased mobility has been reported as a risk factor for falls [37], which is consistent with our present observations. Our present univariate analysis detected a significant association between balance disability and fall history, but the multivariate analysis did not. In a multivariate analysis in which both OLST and TUGT variables were entered separately, we also found that decreased mobility was significantly associated with falls, but balance disability was not significantly associated with falls. This suggests that the association between balance disability and fall history was confounded by other factors. The OLST assesses static balance and is considered a strong predictor of falls [33], but the parameters of the OLST may vary between different testing sessions for the same individuals [38]. In this study, statistically, there was a significant difference in OLST between the positive fall experience group and no fall group, with lower mean values in the positive fall experience group, but the SD was larger in the positive fall experience group than in the no fall group. In other words, we showed that OLST in the positive fall experience group varied widely among individuals. Therefore, OLST may not have been extracted as a factor significantly related to falls in logistics analysis. Further, factors such as fatigue can influence the balance ability [38]. Although static balance is a clinically important factor in the risk of falling, this limited reproducibility of the OLST may have led to the lack of a significant association in the present study.

Our analyses also demonstrated a significant association between depression and fall history. Individuals with more than moderate depressive symptoms are more likely to have a fear of falling than those without depression [39], and such symptoms were reported to be associated with an increased risk of falls in the future [40]. We speculate that a fear of falling may increase in individuals with KOA as their depression worsens, eventually contributing to falls to the same degree as decreased gait and balance functions. In addition, depression is considered a potential mediator of knee pain and falls [41]. Our results indicated a significantly weak positive correlation and correlation with little clinical meaning between them (correlation coefficient; 0.2, (*p*)-value of < 0.0001). It should be noted that knee pain was significantly associated with falls in this study as well, and that depression itself is not only associated with falls, but may also influence falls through the mediation of knee pain. Prior research indicated that psychological factors (e.g., depression) and cognitive factors should be considered when assessing the association between KOA and falls [13]. Our present findings suggest that assessing and treating psychological factors might be important in reducing the risk of falls as well as improving pain.

We also analyzed excluding no OA pattern to account for the effects of depression and decreased mobility not associated with KOA. Our results showed that knee pain, depression, and decreased mobility affected the risk of falling, regardless of the presence or absence of a radiographic KOA.

Our results also demonstrated no significant direct relationship between radiographic KOA severity and fall history. However, it has previously been reported that the risk of falling increases with more severe KOA [20], which contradicts our results. Although KOA is usually diagnosed based on radiographic severity, symptoms of KOA do not always match imaging findings [36, 42]. Similarly, the risk of falls in KOA should not be based on imaging findings alone, but additionally include the patient's clinical symptoms, knee pain, physical function, and psychological factors.

Despite being considered risks for falls in previous studies, gender [12, 18, 19], crepitus [22], and sleeping pill use [26] were not significantly associated with fall history in the present study. it has been indicated that differences in fall rates by gender may reflect differences in lifestyle [43]. Because the subjects in this study were residents of a limited rural region, their lifestyles may not have varied significantly between gender. Crepitus is reported to be poorly understood, especially among the elderly [44]. In this study, self-reports of knee crepitus may have been lower than actual. In addition, since this study was conducted at the same time as the annual health checkups, subjects may have been concerned about their health. Thus, it is possible that there were more healthy subjects and that sleeping pill use was not significantly associated with fall history. Therefore, gender, crepitus, and sleeping pill use may not have been significantly associated with fall history.

Although clinical symptoms and image findings of KOA patients are often the clinical focus, we must not forget to evaluate psychological factors (such as depressive symptoms) and physical function. Regardless of the radiographic KOA severity, the treatment of knee pain and treatment for depression and reduced mobility may reduce the risk of falls. Our present findings provide key points for clinical practice because they show the importance of therapeutic interventions for knee pain, depression, and mobility in preventing falls in the elderly. Further research and intervention studies are required to test our present findings.

Several study limitations must be addressed. First, because this study was a cross-sectional analysis, a causal relationship cannot be determined. Second, we were unable to include polypharmacy, malnutrition, smoking, alcohol consumption, stroke, and Parkinson's disease, which are commonly considered risk factors for falls^26^, in our analysis. Third, we did not investigate the subjects' detailed history of treatment for KOA, which might have affected their symptoms. Fourth, in this study, because plain radiographs of the knee, responses to the questionnaire, and physical function assessment were optional, subjects enrolled in the study differed significantly from those excluded in demographic and clinical variables. Many symptomatic or health-conscious subjects may have participated, possibly biasing the study. The generalizability of the results must be carefully considered. Finally, a subject's fall history may be more excessive or may be insufficient compared to the actual fall history, due to recall bias.

## Conclusions

In conclusion, the results of this study demonstrated that knee pain, decreased mobility, and depression were significantly associated with a history of falling and hence, a risk of future falls. However, the radiographic KOA severity was not significantly associated with fall history. Regardless of the patients' radiographic KOA severity, their risk of falling may be reduced by treating their knee pain, improving their mobility function, and addressing psychological factors.

## Data Availability

The datasets used and/or analyzed during the current study are available from the corresponding author on reasonable request.

## Abbreviations

QOL: Quality of life
KOA: Knee osteoarthritis
LOHAS: The Locomotive Syndrome and Health Outcome in Aizu Cohort Study
BMI: Body mass index
CESD-10: Center of Epidemiologic Studies Depression Scale-10
TUG: Timed Up and Go Test
OLST: One-Leg Standing test
VIF: Variance inflation factor
OR: Odds ratio
